# Hypervalent surface interactions for colloidal stability and doping of silicon nanocrystals

**DOI:** 10.1038/ncomms3197

**Published:** 2013-07-29

**Authors:** Lance M. Wheeler, Nathan R. Neale, Ting Chen, Uwe R. Kortshagen

**Affiliations:** 1 Department of Mechanical Engineering, University of Minnesota, 111 Church Street SE, Minneapolis, Minnesota 55455, USA; 2 National Renewable Energy Laboratory, 15013 Denver West Parkway, Golden, Colorado 80401, USA; 3 Department of Chemical Engineering and Materials Science, University of Minnesota, 421 Washington Avenue SE, Minneapolis, Minnesota 55455, USA

## Abstract

Colloidal semiconductor nanocrystals have attracted attention for cost-effective, solution-based deposition of quantum-confined thin films for optoelectronics. However, two significant challenges must be addressed before practical nanocrystal-based devices can be realized. The first is coping with the ligands that terminate the nanocrystal surfaces. Though ligands provide the colloidal stability needed to cast thin films from solution, these ligands dramatically hinder charge carrier transport in the resulting film. Second, after a conductive film is achieved, doping has proven difficult for further control of the optoelectronic properties of the film. Here we report the ability to confront both of these challenges by exploiting the ability of silicon to engage in hypervalent interactions with hard donor molecules. For the first time, we demonstrate the significant potential of applying the interaction to the nanocrystal surface. In this study, hypervalent interactions are shown to provide colloidal stability as well as doping of silicon nanocrystals.

## Abstract (web summary)

Electrically insulating ligands and doping of colloidal semiconductor nanocrystals continue to be significant challenges for practical nanocrystal-based optoelectronics. Wheeler *et al.* demonstrate a new surface chemistry technique that confronts both of these challenges simultaneously.

## Graphical abstract

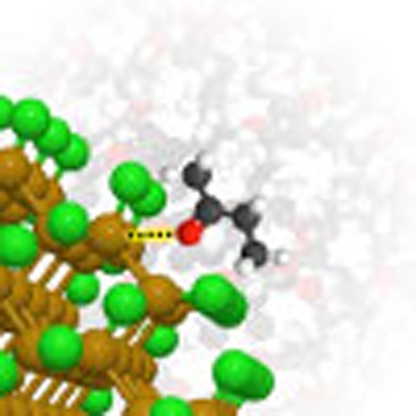

Quantum confinement effects make semiconductor nanocrystals (NCs) particularly interesting at diametres approaching or below the exciton Bohr radius. Processing these materials from colloidal solutions enables roll-to-roll, large-area deposition of NC thin films for optoelectronic devices such as solar cells^1^, light-emitting diodes^2^ and photodetectors^3^. Typical NCs have an exciton Bohr radius on the order of ≤10 nm.

Colloidal stability becomes exceedingly difficult at this scale, as Brownian motion becomes dominant and leads to NC agglomeration^4^. A steric barrier to agglomeration from long-chain organic ligands at the NC surface is typically employed to overcome this difficulty^5^. Ligand-capped NCs are solvated in non-polar solvents and will self-assemble into highly ordered thin films^6^. However, the organic ligands dramatically hinder charge carrier transport in the films^7^,^8^. For the extensively studied metal chalcogenide NCs, this problem is routinely solved during film assembly by chemical exchange of the original ligands for shorter organic^7^ or inorganic^9^ ligands.

Talapin and coworkers^10^,^11^ have pioneered an alternative strategy in which insulating organic ligands are exchanged in solution for conductive molecular metal chalcogenide complexes or metal-free inorganic ions^12^. Conductive films can thus be assembled directly from solution. This strategy represents a second viable mechanism of NC colloidal stability based on electrostatics where the negative charge of the ionic surface species imparts stability in polar solvents.

After deposition of a conductive NC film, control of the optoelectronic properties is paramount to device performance. In bulk semiconductors, this is achieved by doping the controlled addition of atomic impurities into the crystal lattice. This has proven challenging at the scale of confined NCs. Statistical, thermodynamic and kinetic arguments have been employed to rationalize the difficulty of incorporating dopant atoms into NCs^13^, and practical implementation of doped NC films has remained elusive.

We confront the issues of thin-film ligand removal as well as doping of NCs by applying the framework of generalized Lewis acid–base theory^14^ to the silicon (Si) NC surface. Si normally bonds with four atoms to complete its outer shell octet. However, polarization of electron density away from the Si atom renders it Lewis acidic and capable of engaging in pentavalent and even hexavalent bonds^15^,^16^. Though this phenomenon is well known to occur in molecular analogs, for the first time we demonstrate its significant potential when applied to the surface of a NC. This is achieved by first terminating the Si NC surface with chlorine (Cl) to polarize electron density away from Si surface atoms. The Lewis acidic Si–Cl surface groups facilitate hypervalent interactions with hard donor (Lewis base) molecules. In a series of experiments, we show this interaction is responsible for colloidal stability by ruling out previously demonstrated steric^5^ and electrostatic^10^,^11^,^12^ mechanisms. In parallel, the observation of free carrier absorption in the Si NCs upon contact with the donor molecules further validates the interaction as hypervalent in nature and illustrates a new method for reversible molecular doping of NCs.

## Results

### Donor molecules and the Si NC surface

Nonthermal plasma synthesis has proven to be highly successful in the production of group IV NCs when compared with colloidal methods because of the high crystallization energies needed^17^. It also allows for surface chemistry control. In this work, a Cl-terminated surface is achieved via decomposition of SiCl_4_ vapour in the presence of H_2_ in a nonthermal plasma reactor (Supplementary Fig. S1).

**Figure 1 f1:**
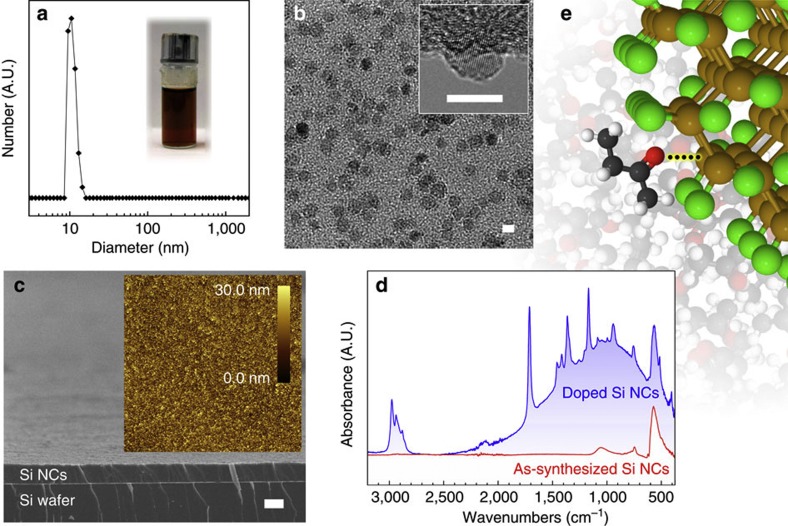
Colloidal stability and doping of Si NCs. Si NCs synthesized without ligands immediately form a concentrated solution when dispersed in hard donor solvents such as 2-butanone (**a**, inset). The process is shown in Supplementary Movie 1. (**a**) DLS spectrum showing a single-particle population of Si NCs in 2-butanone with a diameter of 10 nm, which indicates a solvation shell of 2 nm when compared with transmission electron microscopy (TEM) (**b**). Single-particle solvation is also validated by the two-dimensional ordering observed in TEM. Scale bars, 8 nm (**b**). Scanning electron microscopy demonstrates that device-quality films are attained by drop-casting from these stable colloids (**c**), and scanning probe microscopy (SPM) (inset) shows that films are continuous over large areas with a root mean square roughness of 8.4 nm. Scale bar, 2 μm (**c**). The SPM area is 50 × 50 μm^2^. (**d**) ATR-FTIR spectra depicting as-synthesized Si NCs (red) and Si NCs in the presence of 2-butanone (blue). The broad absorption centered at 1,000 cm^−1^ is attributed to free carrier absorption due to hypervalent interactions at the Si NC surface. (**e**) A cartoon depiction of this hypervalent interaction between a 2-butanone molecule and a Si–Cl group of the Si NC surface.

Addition of an appropriate hard donor solvent to a vial containing Cl-terminated Si NCs yields an optically transparent colloidal solution after slight agitation by simply shaking the vial until a maximum concentration is reached. The process of colloid formation can be seen in Supplementary Movie 1. Dynamic light scattering (DLS) on the Cl-terminated Si NCs solvated in 2-butanone, shown in Fig. 1a, confirms that the Si NCs are unagglomerated with a single-particle population at 10 nm in diametre.

Figure 1b shows transmission electron microscopy images of highly monodisperse 8 nm Si NC cast from a benzonitrile solution. The crystal diameter is in agreement with Scherrer broadening of the X-ray diffraction spectrum (Supplementary Fig. S2) and the 10 nm diameter observed in the DLS spectrum, which includes the solvation layers that surround each NC. Evidence of ordering and lack of agglomeration in the NC monolayer of Fig. 1b also illustrates that NCs are stabilized in solution. As expected for a stable solution^6^, the Si NCs self-assemble into device-quality films. Figure 1c shows a scanning electron microscopy image of a film assembled by drop-casting a 5 mg ml^−1^ solution of Si NCs in 2-butanone onto a gold-coated Si wafer. Scanning probe microscopy shows that films are crack-free and continuous over large areas with a root mean square roughness of 8.4 nm (Fig. 1c, inset).

Solvents that provide colloidal stability also exhibit evidence of doping in the attenuated total reflectance Fourier transform infrared (ATR-FTIR) spectrum upon contact with the Si NCs. An example of this is shown in Fig. 1d. The Si–Cl_*x*_-stretching mode at 575 cm^−1^ is the dominant feature of the infrared spectrum of the as-synthesized Si NCs (Fig. 1d, red). After adding 2-butanone to the Si NCs, additional sharp features are observed due to molecular vibrations of 2-butanone, and a broad free carrier absorption centered at 1,000 cm^−1^ is also present (Fig. 1d, blue).

The observed colloidal stability, as well as free carrier absorption, is indeed dependent on Si NC surface chemistry. For control experiments, we synthesized Si NCs from silane to ensure the production of Si NCs with a fully H-terminated surface^18^. We found H-terminated Si NCs to be insoluble in most solvents, but dilute solutions may be obtained by prolonged sonication, which is consistent with previous work^19^,^20^. Moreover, no free carrier absorption is observed upon interaction with hard donor molecules.

Solvent choice is also important. A number of solvents were used in this study, and colloidal stability was achieved in a variety of donor solvents. A representative list, along with common solvent scales, is compiled in Table 1 (refs 21, 22, 23). This concept will be expanded on in the Discussion section.

### Infrared spectroscopy of Si NC surface interactions

It is clear from observational studies that a Cl-terminated surface is needed to achieve colloidal stability as well as free carrier absorption. Hypervalent interaction between Si–Cl groups with donor molecules is well estabilished in synthetic organosilicon work^24^. Here we employ ATR-FTIR to demonstrate this interaction at the Si NC surface. A cartoon depiction of the interaction between a Si–Cl surface group of a Si NC with the donor carbonyl group of 2-butanone is provided in Fig. 1e.

**Figure 2:**
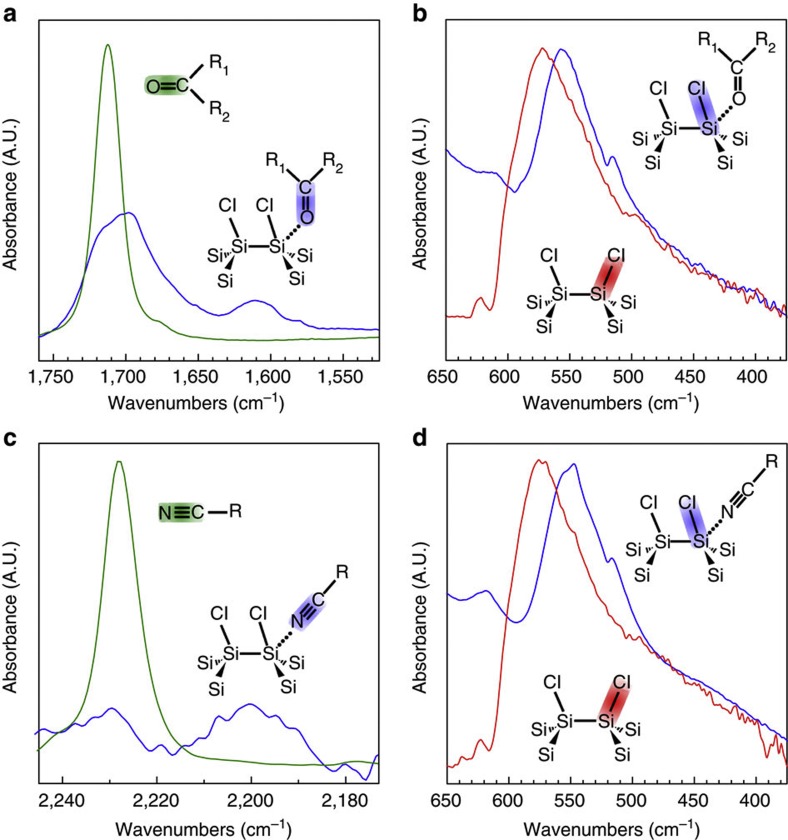
ATR-FTIR characterization of hypervalent interactions. Electron density redistribution due to hypervalent interaction leads to lengthening of the donor group bond as well as the peripheral bonds of the accepting acidic site. This is clearly observed in ATR-FTIR spectra of the Si NC-donor molecule system by red-shifting of the stretching modes of these bonds. (**a**) The carbonyl-stretching region of neat 2-butanone (green) and 2-butanone in the presence of Si NCs (blue). (**b**) The Si–Cl_*x*_-stretching region of as-synthesized Si NCs (red), and the red-shifted stretch in the presence of 2-butanone (blue). The same trend is observed in the nitrile region of the spectrum (**c**) for nitrile solvents interacting with the Si–Cl_*x*_ groups of the Si NC surface (**d**). Bond colour of inset diagrams indicate vibrations observed in the corresponding spectra.

Infrared vibrational modes are well known to be sensitive to changes in electron density and bond length. The carbonyl-25 and nitrile-26 stretching modes have been extensively studied in this regard because of their spectral isolation and sensitivity. Figure 2a shows the infrared spectrum of the carbonyl-stretching region of 2-butanone. The carbonyl stretch of neat 2-butanone is plotted in green with a characteristic frequency of 1,712 cm^−1^. The blue spectrum is taken after a solution of Si NCs is allowed to evaporate onto the ATR crystal. In the presence of Si NCs, the carbonyl peak has broadened and red-shifted by 25 cm^−1^. In addition, a lower-energy satellite peak emerges at 1,610 cm^−1^. Empirical analysis of acid–base interactions by Gutmann^14^ serves as an intuitive guide to understanding the spectral shifting by redistribution of electron density and subsequent change in bond lengths upon Lewis acid–base interaction. Upon interactions, the bond length of the Lewis base (carbonyl) is increased, which polarizes the electron density towards the Lewis acidic site (Si surface atom). The lengthened bond is clearly seen in Fig. 1a as a shift to a lower-energy vibration in the ATR-FTIR spectrum. The satellite peak is likely due to more strongly bound 2-butanone molecules.

The response of the Lewis acidic site (Si surface atom) to this interaction is an increase in the coordination number and a lengthening of the peripheral Si–Cl_*x*_ bonds, which is observed in the infrared spectrum as a red-shift from 575 to 550 cm^−1^ in the Si–Cl_*x*_-stretching modes (Fig. 2b). No other spectral shifting is observed. Secondary bonds also predicted by Gutmann^14^ to be affected, such as the Si-Si or C-C vibrational modes, are difficult to resolve or show no shifting. This is not surprising, as effects due to hypervalency are less significant farther away from the acid–base interaction site.

The ATR-FTIR spectra in Fig. 2c,d show a similar effect of the nitrile group of benzonitrile interacting with Si–Cl_*x*_ groups of the Si NC surface. The nitrile stretch in Fig. 2c is red-shifted from 2,225 to 2,200 cm^−1^. The red-shift is of similar magnitude to that observed in the carbonyl group of 2-butanone. The Si–Cl_*x*_ stretch also shifts from 575 to 550 cm^−1^ (Fig. 2d).

One must be careful in the application of the rules by Gutmann^14^ to the surface of a NC, as structural reorganization of the molecules is typically needed to lower the energy of the newly formed hybrid orbitals. The surface Si atoms of the NC are relatively fixed in comparison, but shifts in electron density are certainly possible. The more rigorous molecular orbital picture is consistent with the empirical rules by Gutmann^14^. Si is able to engage in electron-rich three-centre four electron bonding, which results in a pair of hybrid orbitals that localizes the electron density at the peripheral atoms^15^. Constituent bonds are thus lengthened, which is observed as a red-shift in the ATR-FTIR spectrum. Taken together with Lewis acid–base theory, we conclude that the ATR-FTIR data confirms hypervalent interactions at the Si NC surface. Most importantly, the observations shown for Si NCs in 2-butanone and benzonitrile are generalizable; similar spectral shifting is observed for the many ketones/aldehydes and nitriles investigated.

### Mechanism of Si NC colloidal stability

Colloidal stability is an attractive feature of NC systems, as it enables cost-effective deposition of thin films for optoelectronics. Currently, success in device integration of NCs lies in the ability to chemically exchange insulating ligands for shorter species during film deposition^7^,^9^ or in solution for charged ionic species^10^,^11^,^12^. The key to both of these strategies is ligands that are relatively labile and thus freely exchanged. This is an important distinction to group IV NCs. Although long-chain ligands also provide colloidal stability in group IV NCs^27^,^28^, the surface species are covalently bound, and ligands are kinetically inert to exchange reactions. This feature has precluded significant progress on group IV NC device development when compared with metal chalcogenide NCs. Thus, understanding the mechanism of stability observed in this work will be important for development of colloidal NCs for optoelectronics.

**Figure 3:**
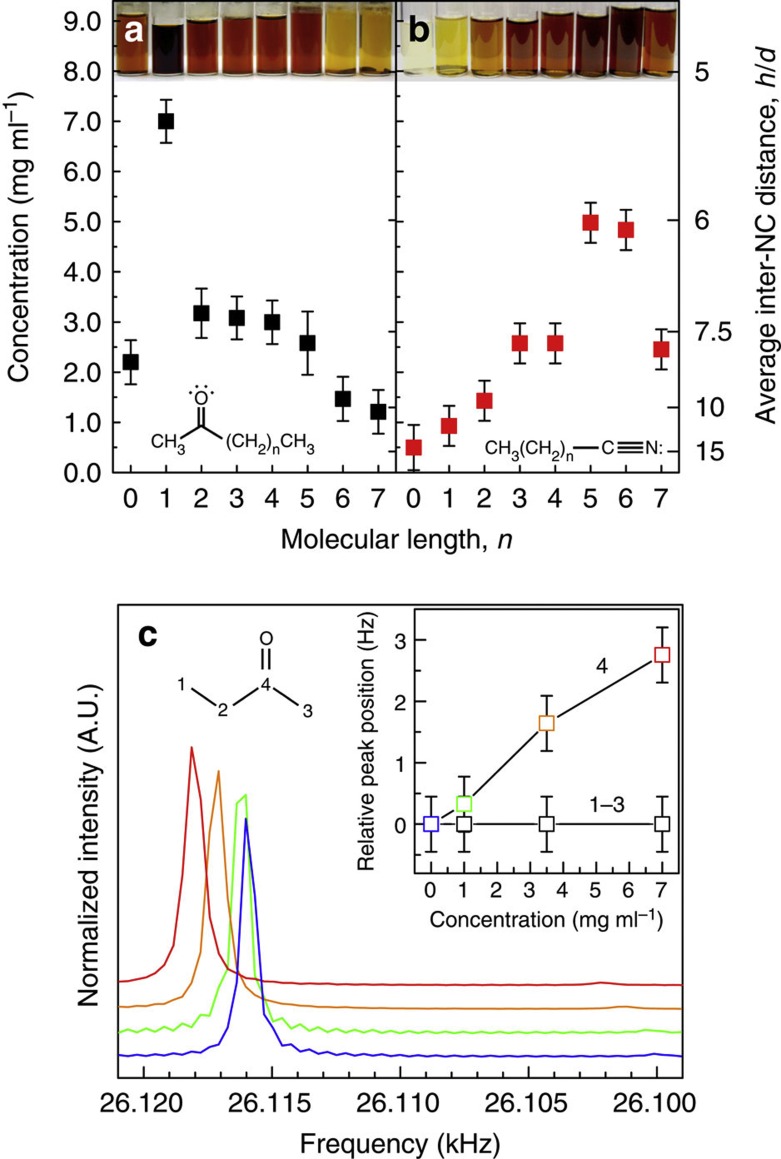
Mechanism of colloidal stability determination. Si NC concentration as a function of molecular length, *n*, for n-alkanones (**a**) and n-alkanenitriles (**b**) as determined by ultraviolet-visible absorption. The right axis is the average inter-NC separation distance. If electrostatics are the dominant mechanism of colloidal stability, then shorter molecules should achieve higher concentrations. If steric effects were dominant, longer molecules are expected to provide more highly concentrated Si NC solutions. Photographs of the solutions are included at the top of the graph. Error bars reflect uncertainty in mass measurements. (**c**) The carbonyl region of the ^13^C NMR spectra of Si NCs stabilized in 2-butanone at concentration of 0, 1, 3.5 and 7 mg ml^−1^. The spectra are normalized and vertically offset for clarity. The downfield shift of the carbonyl peak is due to Si NC surface interactions and resulting interactions in the solvent shells surrounding the NC. Aliphatic carbon peaks (1–3) do not shift relative to neat 2-butanone with increasing Si NC concentration (inset). Error bars correspond to the digital resolution of the spectrometer.

In a series of experiments, we illustrate that hypervalent interactions at the Si NC surface are responsible for colloidal stability rather than the two common mechanisms typically associated with this effect (steric and electrostatic interactions)^29^. We begin by investigating the effects of varying the molecular length of n-alkanones and n-alkanenitriles. Figure 3a,b show Si NC concentration as a function of molecular length for ketones and nitriles, respectively.

If the Si NC colloids are stabilized by steric interactions between the long-chain tails of strongly bound ligands, then longer chain molecules are expected to have a larger steric barrier and provide better stability. However, the data show that Si NC concentration decreases with increasing ketone length (Fig. 3a). Nitriles exhibit a slightly different trend, with Si NC concentration increasing with chain length until heptane- and octanenitrile (*n*=5 and 6, respectively), then decreasing again as chain length is increased further (Fig. 3b). If Si NC colloids are formed from steric interactions between the non-covalently bound solvent molecule, no decrease in stability should be observed upon increasing chain length. Furthermore, adding a typical non-polar solvent used in sterically stabilized solutions, such as hexane, causes Si NCs to quickly flocculate and phase-separate from the stable solution, which suggest that non-polar solvents disrupt the stabilizing forces provided by the weakly bound ketone or nitrile molecules.

We explore the interaction of solvent molecules with the Si NCs using ^13^C nuclear magnetic resonance (NMR). As NMR is a time-averaged technique, solvent dynamics can be probed. Shifts are expected for short-lived interactions (shorter than the NMR signal decay time), whereas a strongly bound molecule would give rise to a second peak. Figure 3c shows the carbonyl region of the ^13^C NMR spectrum at increasing concentrations of Si NCs in 2-butanone. The data show a clear downfield shift of the carbonyl peak with increasing concentration from 0 to 7 mg ml^−1^ (Fig. 2c), which illustrates the fluxional nature of the solvent molecules with the Si NC surface. Peaks corresponding to the aliphatic carbons do not shift (Fig. 2c, inset). This effect is frequently observed in the solvation of ions as well as molecules when dissolved in carbonyl-containing solvents^30^. A downfield shift of the carbonyl carbon establishes direct interaction between the solute and the carbonyl oxygen, as well as intermolecular ordering of dipole moments in the solvation shells surrounding the solute. Though the 2-butanone molecules interact with the Si NC surface, they are not strongly bound.

For electrostatic stabilization, which is typically described by Derjaguin, Landau, Verwey and Overbeek (DLVO) theory, a shorter ketone or nitrile, which have the highest relative permittivity (formerly dielectric constant), will more effectively screen charge. Indeed, high relative permittivity solvents such as water (*ε*=80.1) and formamide (*ε*=111.0) were needed to stabilize NCs after terminating the surface with negatively charged inorganic ligands^12^. If Si NC solubility was dominated by electrostatics, then shorter molecules with a higher relative permittivity should yield more highly concentrated solutions. However, Si NC concentration is highest for ketones (2-butanone, *n*=1) and nitriles (heptanenitrile, *n*=5) with only modest relative permittivities (18.85 and 15.6, respectively) (Since data for heptanenitrile were unavailable, this value was calculated by a linear interpolation between hexanenitrile and octanenitrile).

Additional evidence also supports electrostatics as insignificant. First, the addition of electrolytes to stable solutions should contract any electrical double layer predicted by DLVO theory, which would allow the NCs to agglomerate and fall out of solution. No such effect is observed upon adding 1.0 mg of NaCl to a 1.0-ml solution of 0.5 mg ml^−1^ Si NCs in 2-butanone. Second, Si NCs solvated in ketones show no measurable *ζ*-potential, which is the standard technique to evaluate colloidal charging^31^. Although some nitriles had *ζ*-potentials as high as −25 mV (benzonitrile), this is still below the widely regarded ±30 mV needed for colloidal stability based on electrostatic interactions, as well as the −60 mV observed in the negatively charged molecular metal chalcogenide-terminated NCs^10^. Moreover, a Coulombic model that adapts DLVO to organic solvents would predict a surface potential of ±81.4 mV for 8 nm particles in 2-butanone and ±89.5 mV in pentanenitrile^32^.

It appears that macroscopic models do a poor job of explaining the observed colloidal stability. The average inter-NC separations (*h*/*d*) in our solutions (Fig. 3a,b) are beyond the regime of continuum theory, and non-DLVO forces, such as solvation, will dominate^33^,^34^. The Si NC dispersions have remained stable for over 1 year when kept air-free. Solutions in 2-butanone reach an *h*/*d* value of nearly five NC diametres, and Brownian motion alone would lead to flocculation on the timescale of a few microseconds without a significant barrier to agglomeration. These data suggest that the fluxional hypervalent interactions within these Si NC solutions are indeed the dominant mechanism of stability. Secondary molecule–molecule interactions also likely have a role, as a modest dipole moment (*μ*) is needed in addition to donor strength (Table 1). This explains why 2,3-butanedione does not provide stability, which has similar donor strength to 2-butanone but no dipole moment.

### Surface doping of Si NCs

It is perhaps not surprising that hypervalent NC surface interactions could provide doping effects. From the ATR-FTIR studies, it is clear that additional electron density is being donated from the Lewis base molecule to the Si surface atoms. Similar phenomena have been investigated, and it is often referred to as surface-transfer^35^ or molecular^36^ doping. Experimental studies on porous Si has demonstrated n- and p-type doping with donor (NH_3_) and acceptor (NO_2_) molecules, respectively^37^. A similar effect was demonstrated with Si nanowires in which dopant concentration could be controlled by the partial pressure of the molecular dopant^38^ at the Si surface. Computational studies also validate these experiments^36^,^39^. In each case, Si is reversibly doped by the adsorption of donor or acceptor molecules.

**Figure 4:**
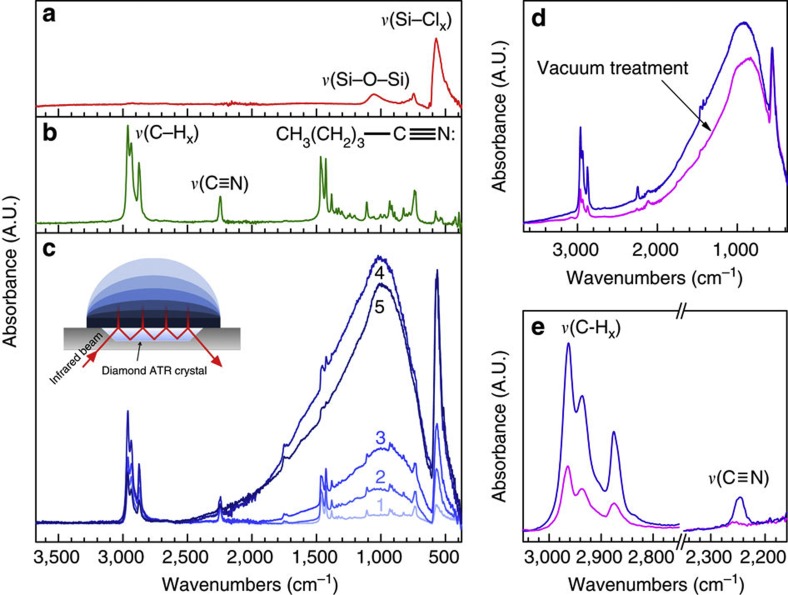
Surface doping of Si NCs. (**a**) ATR-FTIR spectra of as-synthesized Si NCs and neat pentanenitrile (**b**) are provided as reference for **c**, where pentanenitrile is added to Si NCs on an ATR crystal, and spectra are recorded every 20 s. Spectra are not normailized. The spectra are identical to neat pentanenitrile until a broad absorption due to free carriers is observed. The absorption arises from solvent molecules non-covalently bound to the NC surface that effectively dope the NC. As solvent evaporates, the broad infrared absorption band increases in intensity from 1 to 4, then decreases and red-shifts as solvent evaporates from the Si NC surface, and a film is assembled at 5. Much of the remaining solvent can be removed by applying dynamic vacuum to the film (**d**). After 800 s, the absorption intensity of characteristic pentanenitrile vibrational modes, *ν*(C–H_*x*_) and *ν*(C≡N), is decreased by a factor of ~3 (**e**).

In this work, we demonstrate the doping effect by employing ATR-FTIR. Si NCs are first deposited onto an ATR crystal. The spectrum of the as-synthesized Si NCs can be seen in Fig. 4a with the characteristic Si–Cl_*x*_-stretching vibration at 575 cm^−1^. A dense colloid is formed upon addition of pentanenitrile (Fig. 4b) onto the Si NCs. A spectrum is taken every 20 s as the Si NC solution evaporates. The initial spectrum (Fig. 4c1, [Fig f1]) is nearly identical to neat pentanenitrile. The broad absorption then emerges with the Si–Cl_*x*_-stretching vibration of the Si NCs. The infrared absorption intensity increases from 1 to 4 where it reaches a maximum after 640 s of evaporation time. The broad absorption then slowly decreases and red-shifts, as solvent molecules evaporate from the Si NC surface, and a film is assembled at 5.

The broad absorption in Fig. 4c is attributed to free carrier absorption or, in the case of non-interacting NCs, localized surface plasmon resonance. Heavily phosphorus-doped Si NCs were recently shown to exhibit localized surface plasmon resonance in this mid-infrared region^40^. Applying similar analysis, the absorption feature centered at 1,000 cm^−1^ corresponds to an approximate doping concentration of 10^20^ cm^−3^. This high doping level is consistent with the high surface-area-to-volume ratio of the 8-nm NCs.

The surface doping effect appears reversible. The broad absorption peak red-shifts as surface molecules evaporate from spectrum 4 to 5 in Fig. 4c. The Si NC solution was allowed to evaporate for roughly 12 h. This spectrum is shown in Fig. 4d (blue). Applying dynamic vacuum (roughly 10^−2^ torr) to the sample for 800 s decreases the intensity and red-shifts the free carrier absorption. At the same time, the absorption intensity of the C–H_*x*_ and C≡N vibrations associated with surface molecules decrease by a factor of three (Fig. 4e), which indicates that the dopant effect is indeed due to hypervalent interactions at the Si NC surface.

The ability to remove surface molecules is also clear from thermogravimetric analysis (Supplementary Fig. S3). In fact, attempt to corroborate the observed ATR-FTIR shifts with X-ray photoelectron spectroscopy was not possible because signal from the solvent was not present. This is presumably due to solvent evaporation in ultra-high vacuum (10^−9^ torr), which apparently removes surface-bound molecules. These observations are consistent with the fluxionality of 2-butanone molecules observed by ^13^C NMR.

### Electrical characterization of Si NC thin films

**Figure 5:**
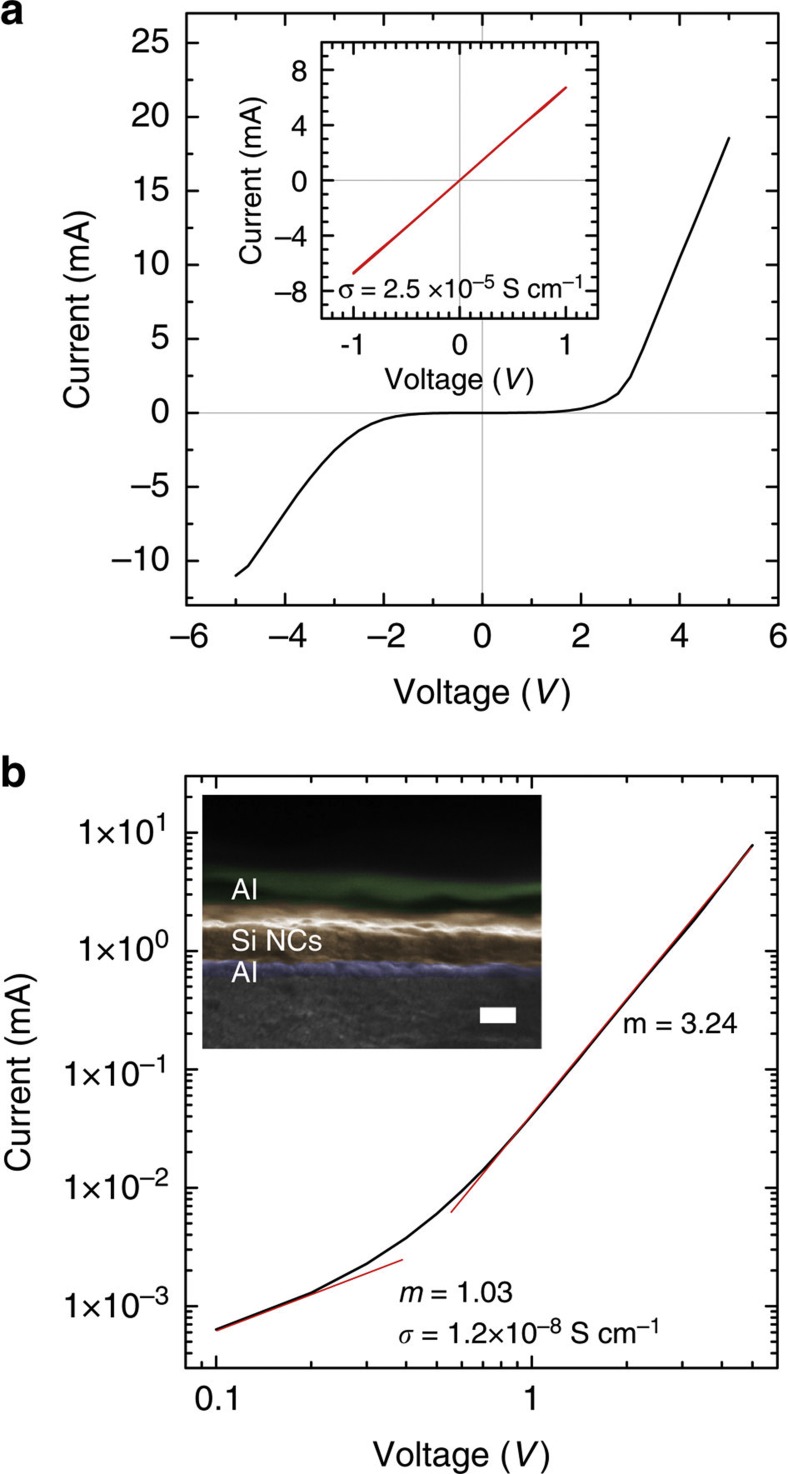
Si NC thin-film device characterization. (**a**) The inset is a typical curve of a Al/Si NC/Al device in which the Si NC film was cast from benzonitrile and allowed to naturally evaporate. Without annealing or application of voltages >1 V, Si NC films show highly conductive ohmic transport exhibiting dark conductivities as high as 10^5^ S cm^−1^. We believe this is due to surface doping effects. After applying voltages >1 V or annealing the film, the films are no longer ohmic. A typical curve is shown in **a**. (**b**) A log–log plot of the device characteristics of a film cast from benzonitrile and vacuum annealed at 200 °C for 12 h at 10^−2^ torr. Fitting the log–log current-voltage curves shows that films exhibit ohmic transport at >0.2 V corresponding to a dark conductivity of 10^8^ S cm^−1^. Space-charge-limited current (*m*=3.24) is observed at higher voltages. The inset is a colourized scanning electron microscopy image showing the device cross-section.

Initial efforts were made to demonstrate the viability of Si NC films for electronic devices by fabricating a simple Al/Si NC/Al vertical structure. Device areas of 1 and 4 mm^2^ show consistent behaviour. Figure 5 shows examples of the typical behaviour observed. Without heat or vacuum treatment, the Si NC films were highly conductive at voltages <1 V and exhibit dark conductivites as high as 10^−5^ S cm^−1^ (Fig. 5a, inset). We believe this is due to surface doping effects, which indicates incomplete removal of bound solvent molecules. Films also showed little response to temperature when varied from 298 to 80 K, which also indicates extrinsic conduction.

After applying voltages up to 5 V (Fig. 5a), the films show non-ohmic behaviour. The same behaviour is observed for films that are annealed or treated with vacuum, which suggests that these treatments disrupt molecular doping effects. Figure 5c shows a log–log plot of a film cast from benzonitrile and vacuum annealed at 200 °C for 12 h at 10^−2^ torr. The slope (*m*) of the log–log curve is 3.24 at voltages >0.2 V, which indicates space-charge-limited current. The films still exhibit ohmic (*m*=1) transport at voltages <0.2 V corresponding to a dark conductivity of 10^−8^ S cm^−1^. This is consistent with the intrinsic conduction observed in a recent report of undoped Si NC films deposited by aerosol impaction^41^. We are currently working to manipulate film-casting conditions to improve consistency of these initial investigations.

## Discussion

**Figure 6:**
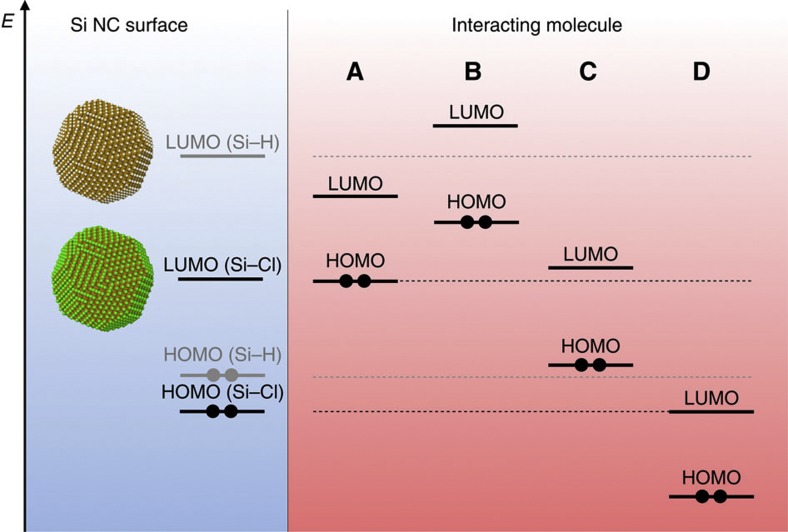
Molecular orbital picture. The Cl termination of the Si NC surface effectively lowers the LUMO energy in comparison with the H-terminated Si NC surface (that is, the Si–Cl group is more Lewis acidic). Molecule A represents a molecule with favourable energetic alignment for hypervalent interactions. In this study, we find ketones, aldehydes and nitriles to fit this criteria. B molecules are strong Lewis base donors such as dimethylformamide and pyridine. These molecules also donate electron density but to an extent where the Si–Cl surface is not preserved. C molecules are chemically inert molecules such as hydrocarbon and chlorinated hydrocarbon solvents, and D molecules represent protic solvents, such as alcohols, that contain acidic hydrogens, which hydrolyse the Si–Cl group. HOMO, highest occupied molecular orbital.

To elucidate the observed interactions of donor molecules with the Si NC surface, we invoke a simple molecular orbital theory model (Fig. 6). The lowest unoccupied molecular orbital (LUMO) of the Si-H groups is too high to afford a favourable interaction with donor molecules. Electron density is polarized away from the Si surface atoms with Cl termination, which effectively lowers the LUMO of the Si NC surface^42^. The LUMO of Cl-terminated Si NCs is now at an accessible level to interact with the highest occupied molecular orbital of donor molecules, whereas the LUMO of H-terminated Si NCs remains inaccessible. This is consistent with our observations that hypervalent interactions, and subsequent colloidal stability and free carrier absorption, are only realized in Cl-terminated Si NCs.

Interacting molecule ‘A’ in Fig. 6 represents molecules such as ketones, aldehydes and nitriles that have a highest occupied molecular orbital near the same energy as the Si–Cl LUMO and are thus able to favourably interact with the Si–Cl surface groups. Electron density is donated from the donor group of the molecule to the Si surface in the form of a hypervalent interaction. This leads to the characteristic shifting in the ATR-FTIR peaks previously discussed.

Interacting molecule ‘B’ of Fig. 6 is a strong Lewis base. This includes common oxygen donors such as dimethylformamide and DMSO, as well as nitrogen donors such as pyridine and triethylamine. Charge transfer should occur with these molecules as well, but, in general, acid–base interactions exist on a continuum between hypervalent states and ionized states^15^. As the strength of the donor increases and the number of hypervalent bonds increases, peripheral bonds (Si–Cl) become more polarized and unstable, which leads to the formation of an inner sphere ionic complex. This leads to enhanced susceptibility to hydrolysis^43^. Although the more sterically hindered donor nitrogen of pyridine shows evidence of hypervalent interaction (Supplementary Fig. S4), hydrolysis with trace amounts of water in a N_2_-purged glovebox is observed in the ATR-FTIR spectrum upon interaction with strong donors such as dimethylformamide (Supplementary Fig. S5).

Finally, molecule ‘C’ of Fig. 6 represents non-polar, chemically inert solvents such as hexane and benzene, and molecule ‘D’ represents solvents that are yet to be discussed, which are protic solvents that possess an acidic hydrogen. These molecules hydrolyze the Si–Cl group.

It is clear that donor strength is an important parameter for hypervalent interaction with the Si NC surface from this analysis. Table 1 provides a variety of solvent scales that reflect physical properties (relative permitivity, dipole moment, polarizability and so on) as well as basicity (donor strength) of investigated solvents. *pK*_BH_, Gutmann donor number (DN) and *−*Δ*H*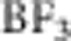 are common basicity scales that reflect the relative strength of interaction between a Lewis base and a standard Lewis acid. Though the scale has seen criticism^21^, antimony pentachloride is a sterically hindered molecule that is a reasonable analog to the Si–Cl surface. It appears that DNs between 13 and 17 kcal mol^−1^, which correspond to ketones/aldehydes and nitriles, yield favourable interaction with the Si NC surface. Higher DNs result in removal of Cl^−^ from the Si surface, and lower DNs do not noticeably interact. These trends are comparable for the remaining basicity scales. In fact, even small differences in DN between typical ketones and nitriles are noticeable (Supplementary Fig. S6).

In conjunction with experimental evidence, this model has important implications for many NC systems. By modulating the Lewis acidity of the NC surface, we have demonstrated hypervalent interactions as an avenue for colloidal stability and charge-transfer doping. Hypervalency is not limited to Si. In fact, heavier group 14 elements (Ge, Sn and Pb) should accommodate hypervalent interactions better than Si. We believe that this is the mechanism previously observed in the colloidal stability^44^ of Cl-terminated Ge NCs. Boron and all main group elements below the third period of the periodic table are well known to engage in hypervalent interactions as well. This includes widely studied IV–VI compounds (PbS and PbSe) as well as emerging III–V NC materials.

The results presented here have the potential to tackle two fundamental challenges in achieving practical application of colloidal NCs for optoelectronic devices. Hypervalent surface interactions observed at the Si NC surface has led to a novel mechanism of colloidal stability as well as a surface doping effect. This is achieved by terminating the Si NC surface with Cl, which leads to an acidic Si surface atom that can favourably interact with a particular class of donor solvents. We are able to understand and validate these findings with the application of a simple molecular orbital model, which we believe can be extended to encompass a variety of NC material systems.

## Methods

### Si NC synthesis

Cl-terminated Si NCs were synthesized by flowing 30 sccm of argon, 20 sccm of H_2_ and 4 sccm SiCl_4_ into an evacuated plasma reactor^17^ and applying a nominal radio frequency power of 200 W at 13.56 Hz to a pair of ring electrodes separated by 1 cm and mounted on an alumina tube (1.9 cm outer diameter, 1.27 cm inner diameter). The pressure in the reactor was 733 Pa. See Supplementary Information for a more detailed description.

### Colloid formation

The chlorinated surface is quite reactive to ambient conditions, and all processing is done air-free. Si NCs are collected by impacting them onto a substrate downstream of the plasma. The Si NCs are transferred from the reactor in a vacuum-component assembly pressurized with argon and further processed on a Schlenk line or in an inert-atmosphere glovebox. Si NCs were transferred into an ampule and capped with a septum in the glovebox. The capped ampule is removed from the glovebox, and a solution is formed by transferring solvent to the ampule via cannula. The nitrogen atmosphere is removed, and the ampule is flame-sealed before returning to the glovebox for manipulation and spectroscopic characterization.

### Solvents

The following solvents were purchased from Sigma Aldrich (purity and grade are included if available): acetone (≥99.9%, high performance liquid chromatography (HPLC)), 2-butanone (≥99.7%, HPLC), 2-pentanone (99.5%, HPLC), cyclohexanone (99.8%), acetophenone (>99.0%, GC), benzaldehyde (≥99%), 2,3-butanedione (97%), trioctylphosphine oxide (99%), trioctylphosphine (97%), acetonitrile (≥99.9%, HPLC), propionitrile (99%), butyronitrile (≥99%), valeronitrile (99.5%), hexanenitrile (98%), heptyl cyanide (97%), nonanenitrile (98%), benzonitrile (99.9%, HPLC), diethyl ether (≥99%, anhydrous), tetrahydrofuran (≥99.9%, anhydrous), 1-methyl-2-pyrrolidone (>99%, HPLC), dimethylformamide (99.8%, anhydrous), dimethylsulphoxide (≥99.9%), pyridine (99.8%, anhydrous), triethylamine (99%), hexane (>97.0%, HPLC), benzene (≥99.9%, HPLC), 1-chloropropane (98%), and 1,2 dichlorobenzene (99%). The following solvents were purchased from Alfa Aesar: 2-hexanone (98%), 2-heptanone (99%), 2-octanone (98%), 2-nonanone (98.%), 2-decanone (97%) and octanenitrile (97%). Solvents were stored on a Shlenk line over 3Å molecular sieves for at least 24 h after three freeze–pump–thaw cycles were performed to remove residual water and oxygen.

### Film formation

Thin films of Si NCs were assembled by drop-casting onto a Au-coated Si wafer, placing a small funnel over the film to slow evaporation. The scanning probe microscopy topology image was obtained on a Digital Instruments Nanoscope operating in tapping mode with a scan rate of 0.4994 Hz and 512 lines per image.

### DLS and *ζ*-potential

DLS spectra and electrophoretic mobility were evaluated on a ZetaPALS (Brookhaven Instrument) using phase-angle light scattering with varied conditions between 50 and 200 V at 2 Hz. *ζ*-potential values were evaluated from the electrophoretic mobility by applying Henry's equation at the Smoluchowski limit^31^. Relative permittivities were obtained from the Landolt–Börnstein Database^22^.

### Calculations

The average inter-NC distance, *h*, is derived within the close-packing model of uniform spheres: *h*/*d=*(*π*/(3
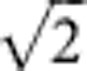
*φ*))^1/3^−1, where we normalize by the NC diametre, *d*, and *φ* is the volume fraction of Si NCs to solvent. We assume bulk density of Si. Calculation of the half-life of the NC solution follows from the Smoluchowski theory of diffusion-limited (perikinetic) aggregation. The derivation can be found in many textbooks^29^ and assumes uniform Brownian spheres under no external fields. We have: *t*_1/2_=*πμd*^3^(8*k*_B_*Tφ*) where *μ* is the viscosity of the medium, *k*_B_ is the Boltzmann constant and *T* is temperature. The half-life of the solution drops steeply with particle size, which illustrates the difficulty of stabilizing particles with diametres as small as the NCs investigated in the main text.

### Colloid concentration

Ultraviolet-visible absorption of centrifuged Si NC solutions was performed on a Cary 5E ultraviolet-visible spectrophotometer. Centrifugation was done at 4,000 r.p.m. for up to 30 min. After centrifugation, optically transparent solutions indicate insignificant scattering, and the Beer–Lambert law (*A*=*εlc*) is employed to determine mass concentration from absorption by integrating the absorption spectra (Supplementary Fig. S7) between 550 and 800 nm. The absorbance, *A*, is linearly dependent on concentration, *c*. The path length through the solution, *l*, and the extinction coefficient, *ε*, are constants. *A* was determined by integrating under the absorbance curve. The absorbance of 5.6 mg of Si NCs in 1.25 ml of 2-butanone, *A*_b_, did not change after centrifugation, so the concentration, *c*_b_, was known. It was used to determine the unknown concentration, *c*_o_, of other solutions relative to 2-butanone. After subtracting the background solvent, the concentration of other solutions is obtained from: *A*_*o*_/*A*_b_=*c*_o_/*c*_b_.

### ^13^C NMR

Samples were prepared by transferring Si NC solution to an evacuated quartz NMR tube and flame-sealing the tube. ^13^C NMR spectra were obtained on a Bruker Avance III 500 MHz spectrometer equipped with a BBFO Smart Probe by locking onto a D_2_O capillary at ambient temperature. 1,024 scans were obtained at 125 MHz, with a 29,761.9 Hz sweep width, 30° pulsewidth, 1.1 s acquisition time and 2.0 s relaxation delay.

### ATR-FTIR

ATR-FTIR experiments were done on a diamond ATR crystal using a Bruker Alpha FTIR spectrometer inside a N_2_-atmosphere glovebox. Spectra were typically collected by averaging 24 scans at 2 cm^−1^ resolution.

### Si NC thin-film device fabrication

Films of Si NCs were spin-cast at 1,500 r.p.m. for 1 min on a glass substrate with 40 nm pre-deposited aluminum electrode. Si NCs were cast from either 2-butanone or benzonitrile and allowed to dry at atmospheric pressure and under 10^−7^ torr vacuum for up to 12 h. Typical film thickness measured in scanning electron microscopy was between 100 and 150 nm. The 1- and 4-mm^2^ vertical devices were completed by evaporating a 100-nm top aluminum contact. Films are annealed and vacuum-treated under a halogen lamp using an in-house fabricated annealing chamber with 10^−2^ torr vacuum.

## Author contributions

L.M.W. designed experiments, analysed data, developed Si NC synthetic methods, performed DLS, *ζ*-potential measurements, ultraviolet-visible spectroscopy and ATR-FTIR spectroscopy, prepared ^13^C NMR and scanning probe microscopy samples, compiled table data, fabricated Al/Si NC/Al devices, developed molecular orbital model and wrote the manuscript. N.R.N. assisted in experimental design, data interpretation and manuscript editing. T.C. contributed transmission electron microscopy images, performed current–voltage measurements of Al/Si NC/Al devices and assisted in current–voltage data analysis. U.R.K. contributed to experimental design, data interpretation and manuscript editing.

## Additional information

**How to cite this article:** Wheeler, L. M. *et al.* Hypervalent surface interactions for colloidal stability and doping of silicon nanocrystals. *Nat. Commun.* 4:2197 doi: 10.1038/ncomms3197 (2013).

## Supplementary Material

Supplementary Figures and Reference Supplementary Figures S1-S7 and Supplementary Reference

Supplementary Movie 1 Demonstration of Si NC surface chemistry effects on colloidal stability.

## Figures and Tables

**Table 1:** Solvent characteristics for colloidal stability and doping of Si NCs.

**Solvent**	*ε*_r_*	***μ***†	***E*_T_ (30)**‡	π^*^§	***pK*_BH_**||	**DN**¶	**−Δ*H*** 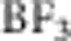 #	**Stability**
*Ketones/aldehydes*								
Acetone (*n*=0)	21.36	2.88	42.2	0.71	1.18	17.03	76.03	Yes
2-Butanone (*n*=1)	18.85	2.78	41.3	0.67	1.22	17.43	76.07	Yes
2-Pentanone (*n*=2)	15.38	2.74	41.1	—	1.17	17.50	76.19	Yes
2-Hexanone (*n*=3)	14.56	2.69	40.1	—	1.18	—	74.60^21^	Yes
Cyclohexanone	16.02	3.06	39.8	0.76	1.39	17.79	76.36	Yes
Acetophenone	18.18	3.05	40.6	0.90	1.11	15.00	74.52	Yes
Benzaldehyde	17.85^22^	—	—	—	0.78	—	74.88	Yes
2,3-Butanedione	4.71^22^	0.00	—	—	—	—	—	No

*Phosphine oxides*								
TOPO/TOP**	—	—	—	—	—	—	—	Yes
								
*Phosphines*								
TOP**	—	—	—	—	—	—	—	No

*Nitriles*								
Acetonitrile (*n*=0)	35.94	3.95	45.6	0.75	0.91	14.60	60.39	Yes
Propionitrile (*n*=1)	28.86	4.04	43.6	0.71	0.93	16.10	60.96	Yes
Butyronitrile (*n*=2)	24.56	4.07	42.5	0.71	0.93	16.60	61.18	Yes
Valeronitrile (*n*=3)	20.03	4.12	42.4	—	—	—	60.75^21^	Yes
Hexanenitrile (*n*=4)	17.26	3.48	—	0.90	0.89	—	—	Yes
Benzonitrile	25.20	4.28	46.7	—	0.80	13.00	55.44	Yes

*Ethers*								
Diethyl ether	4.42	1.11	34.5	0.27	1.01	19.20	78.77	No
Tetrahydrofuran	7.47	1.69	37.4	0.58	1.28	20.50	90.40	No

*Other oxygen donors*								
1-Methyl-2-pyrrolidone	32.58	3.75	42.2	0.92	2.19	27.30	112.56	No
Dimethylformamide	37.06	3.79	43.2	0.88	2.10	26.60	110.49	No
Dimethylsulfoxide	46.71	3.96	45.1	1.00	2.54	29.80	105.34	No

*Other nitrogen donors*								
Pyridine	13.22	2.21	40.5	0.87	1.86	34.00	128.08	No
Triethylamine	2.45	0.72	32.1	0.14	2.13	31.70	135.87	No

*Hydrocarbons*								
Hexane	1.89	0.00	31.0	−0.08	—	0.00	—	No
Benzene	2.27	0.00	34.3	0.59	−0.49	0.10	—	No

*Chloronated hydrocarbons*								
1-Chloropropane	8.53	2.06	37.4	—	−0.30	—	—	No
1,2-Dichlorobenzene	10.36	2.53	38.0	0.80	—	3.00	—	No††

Values are compiled from Abboud and Notari^23^ unless otherwise noted. The *n* value listed for ketones and nitriles corresponds to the molecular length in Fig. 3a.

^*^ε_r_ is relative permittivity (formerly dielectric constant).

^†^*μ* is the modulus of the molecular dipole moment (*D*).

^‡^*E*_T_ (30) is a common polarity scale (kcal mol^−1^).

^§^π* is a common polarizibility scale (kcal mol^−1^).

^||^*pK*_*BH*_ is a basicity scale based on hydrogen bond formation of a donor molecule with an OH group (kJ mol^−1^). Compiled from ref. Laurence and Gal^21^.

^¶^DN is the Gutmann donor number, a basicity scale defined as the negative enthalpy change upon interaction of donor molecule with SbCl_5_ (kcal mol^−1^).

^#^−Δ*H*_*BF*3_ is a basicity scale based on the negative enthalpy change upon interaction of a donor molecule with BF_3_ (kJ mol^−1^).

^**^1 ml of 1.9 μM trioctylphosphine oxide (TOPO) in trioctylphosphine (TOP) was added per milligram of Si NCs.

^††^Si NCs appear to stabilize but can be centrifuged or filtered out.
