# Supplementary material for: Hypervalent surface interactions for colloidal stability and doping of silicon nanocrystals
Source: Nat Commun. 2013 Jul 29;4:2197. doi: 10.1038/ncomms3197 (PMC3731669; doi:10.1038/ncomms3197)
Supplement: Supplementary Figures and Reference — Supplementary Figures S1-S7 and Supplementary Reference [file ncomms3197-s1.pdf]

Hypervalent surface interactions for colloidal stability and doping of silicon nanocrystals

Lance M. Wheeler[1], **Nathan R. Neale**[2], **Ting Chen**[3] and **Uwe R. Kortshagen**[1]

[1] *Department of Mechanical Engineering, University of Minnesota, 111 Church Street SE, Minneapolis, Minnesota 55455, United States*
[2] *National Renewable Energy Laboratory, 15013 Denver West Parkway, Golden, Colorado 80401, United States*
[3] *Department of Chemical Engineering and Materials Science, University of Minnesota, 421 Washington Avenue SE, Minneapolis, Minnesota 55455, United States*

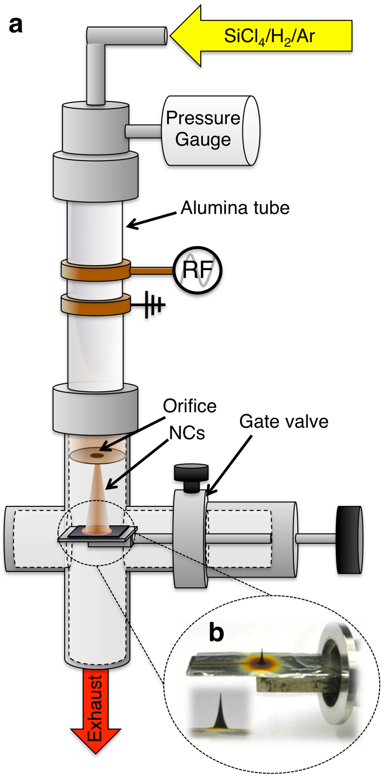

Supplementary Figure S1: a, Si NCs are synthesized in a flow-though plasma reactor. 30 standard cubic centimeters per minute (sccm) of argon, 20 sccm of H_2, and 4 sccm of silicon tetrachloride vapor are injected into a 1.90 cm outer diameter, 1.27 cm inner diameter alumina tube. A plasma is ignited by applying 200 W of nominal radiofrequency power through a matching network at 13.56 MHz to a pair of ring electrodes separated by 1 cm. The precursors are dissociated, and the products chemically nucleate to form small crystallites. The pressure in the reactor is 733 Pa. Pressure and flow rates determine the residence time and, thus, the eventual size of the Si NCs. Sizes ranging from 3 nm to 18 nm have been produced. An orifice is placed downstream of the plasma. After synthesis, Si NCs are accelerated through the orifice and collected using a gas-phase impaction technique[46], which allows for deposition onto a variety of substrates. The NCs form a dense, high aspect-ratio tower (**b**).

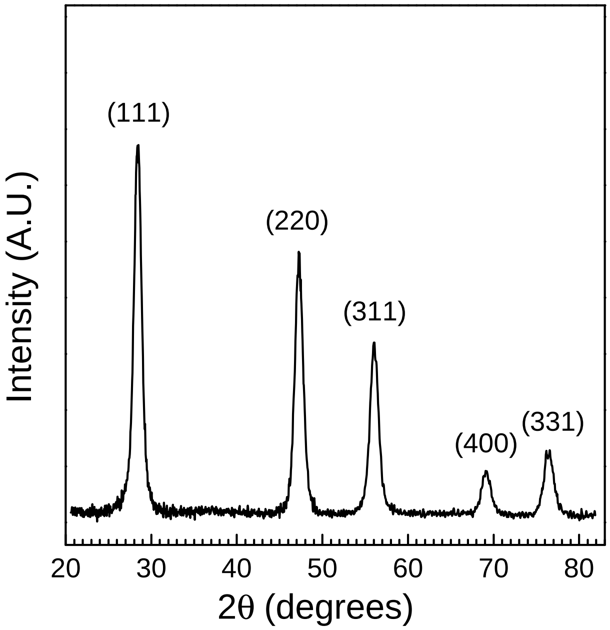

Supplementary Figure S2: Si NCs used in the main text are 8 nm in diameter as determined by Scherrer broadening of the X-ray diffraction pattern. This is consistent with transmission electron micrographs and dynamic light scattering data.

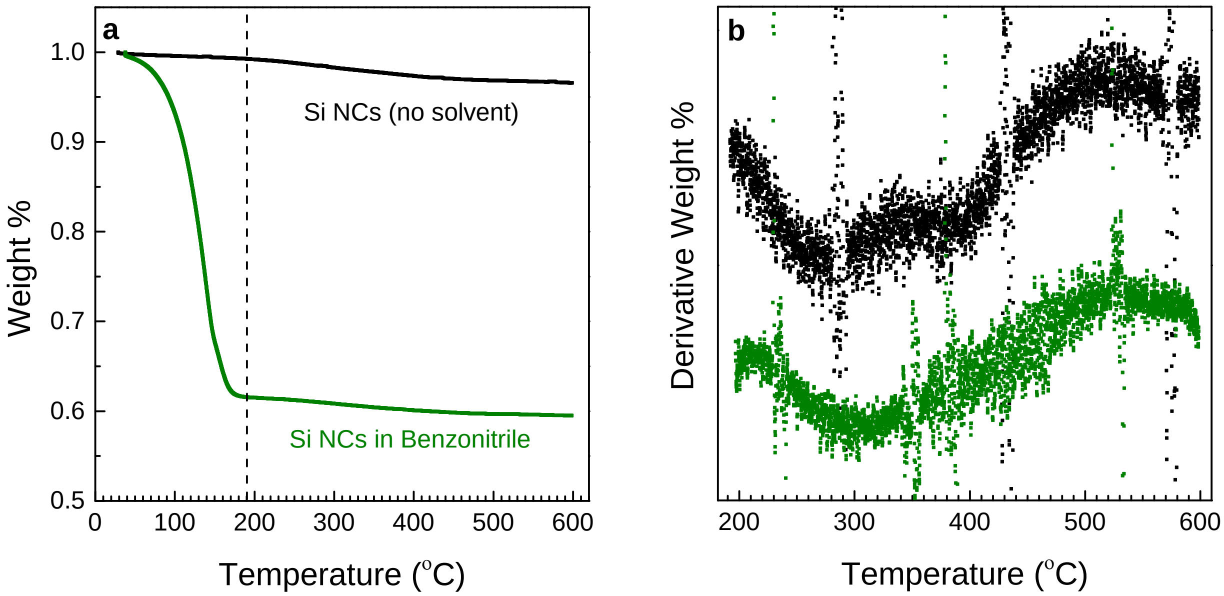

Supplementary Figure S3: To demonstrate removal of dispersing solvent after film formation, we employed thermogravimetric analysis (TGA). **a**, Normalized weight as a function of temperature for 12.98 mg Si NCs transferred directly to a TGA pan (black). Benzontrile was added to a second TGA pan, and Si NCs were added to form a dense colloid. The solvent was allowed to naturally evaporate under N_2 purge. After 12 hours the sample weight remained constant 36.81 mg to indicate the free dispersing solvent molecules had evaporated. This sample is the green spectrum in (**a**). The experiments were performed in a N_2 atmosphere. A ramp rate of 10 °C/min was used. The sample of Si NCs solvated in benzontrile lost roughly 40% of its weight as it approached the boiling point of benzonitrile at 190°C (dashed vertical line). **b**, The derivative spectrum shows the weight loss behavior of the Si NCs solvated in benzonitrile (green) after the solvent has evaporated (200°C) is nearly identical to the as-synthesized Si NCs (black).

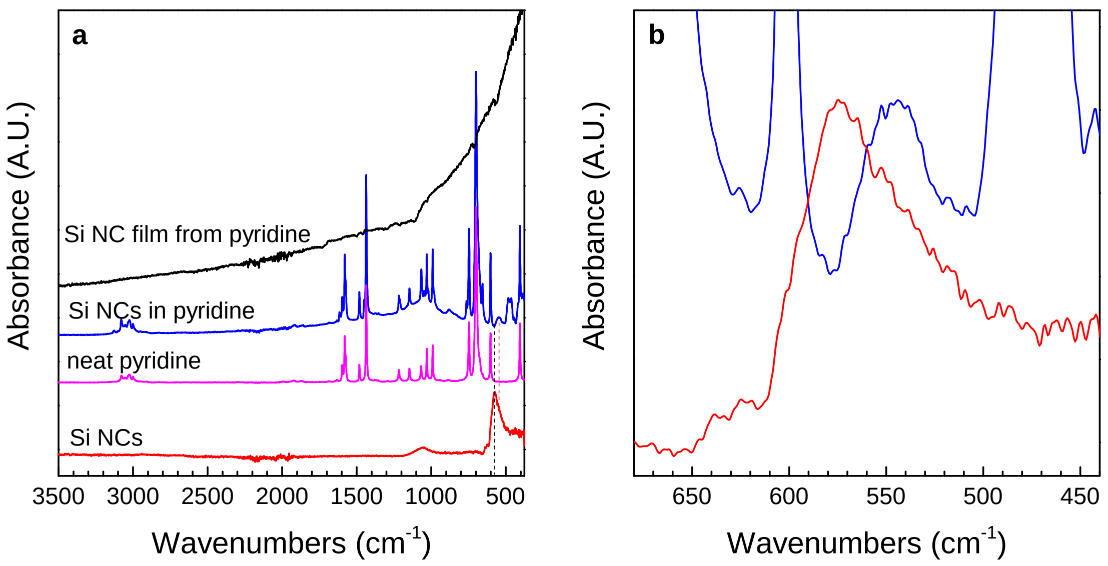

Supplementary Figure S4: **a**, Attenuatated total reflection-Fourier transform infrared (ATR-FTIR) spectra of as-synthesized Si NCs (red). Neat pyridine (magenta) is added to the Si NCs on the ATR crystal (blue). The resulting film (black) shows few molecular vibration peaks but has characteristic ν^3 (ν=frequency) dependence of free carrier absorption. Spectra are offset for clarity. **a**, Si-Cl$_x$ region of the spectrum where a 25 cm^{-1} red-shift in the solvated NCs (red) can be observed in comparison to the as-synthesized Si NCs (blue).

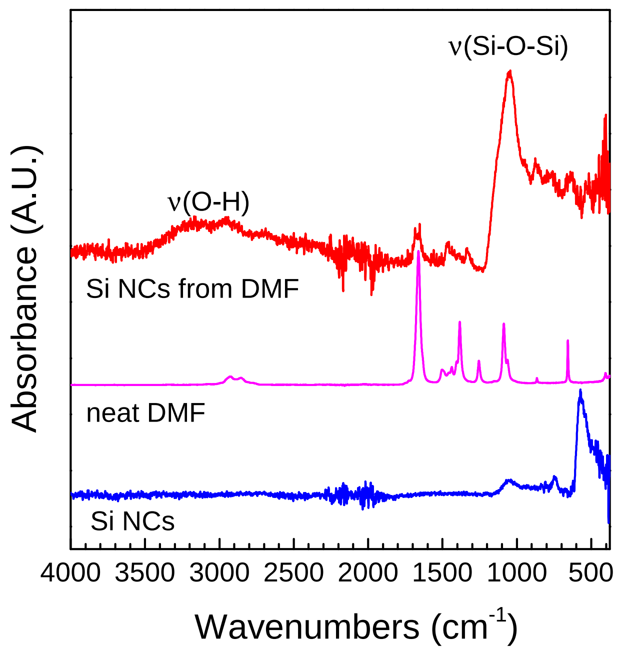

Supplementary Figure S5: a, ATR-FTIR spectra of as-synthesized Si NCs (blue) and neat dimethylformamide (magenta). After the addition of dimethylformamide to the Si NC sample, the resulting film (red) is oxidized by trace amounts of water in the N_2-purged glovebox. This is apparent from the Si-O-Si vibrational mode that appears at 1090 cm^{-1} and the broad O-H absorption centered at 3200 cm^{-1}. Spectra are offset for clarity.

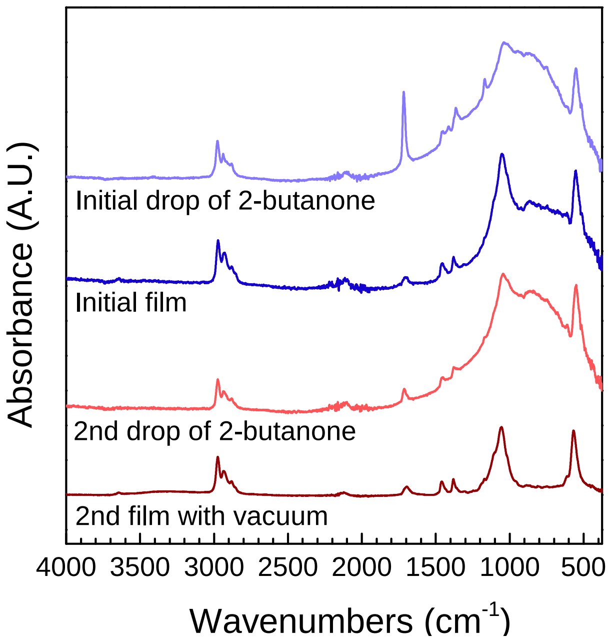

Supplementary Figure S6: Si NCs are dispersed in 2-butanone (light blue) and the solvent is allowed to evaporate to form a Si NC film on the ATR crystal (dark blue). There is a broad absorption due to free carriers. The Si-O-Si peak is indicative of hydrolysis from trace water in the N_2 glovebox. A second drop is added to the same film (light red) and allowed to evaporate. The free carrier absorption peak returns (light red). The film is then put under dynamic vacuum (10^{-2} Torr) to remove solvent and demonstrate reversibility of doping effect. The free carrier absorption peak is no longer visible (dark red). Spectra are offset for clarity.

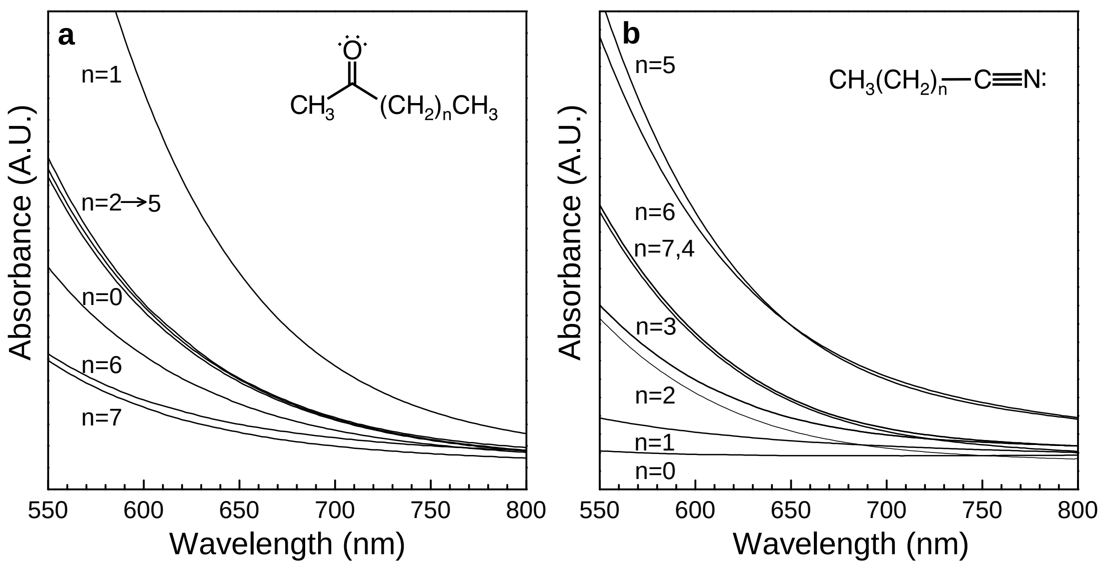

Supplementary Figure S7: Ultraviolet-visible absorption spectra of n-alkanones (**a**) and n-alkanenitriles (**b**) with increasing molecular length, n. Absorbance of NC solutions were measured in sealed vials. The concentration of the Si NC solutions was determined by applying the Beer-Lambert law.

Supplementary References

46. Holman, Z. & Kortshagen, U. A flexible method for depositing dense nanocrystal thin films: impaction of germanium nanocrystals. *Nanotechnology* **21**, 335302 (2010).
